# Spatial and Temporal Heterogeneity in High-Grade Serous Ovarian Cancer: A Phylogenetic Analysis

**DOI:** 10.1371/journal.pmed.1001789

**Published:** 2015-02-24

**Authors:** Roland F. Schwarz, Charlotte K. Y. Ng, Susanna L. Cooke, Scott Newman, Jillian Temple, Anna M. Piskorz, Davina Gale, Karen Sayal, Muhammed Murtaza, Peter J. Baldwin, Nitzan Rosenfeld, Helena M. Earl, Evis Sala, Mercedes Jimenez-Linan, Christine A. Parkinson, Florian Markowetz, James D. Brenton

**Affiliations:** 1 Cancer Research UK Cambridge Institute, University of Cambridge, Cambridge, United Kingdom; 2 Department of Oncology, Hutchison/MRC Research Centre, University of Cambridge, Cambridge, United Kingdom; 3 Cambridge University Hospitals NHS Foundation Trust, Cambridge, United Kingdom; 4 NIHR Cambridge Biomedical Research Centre, Cambridge, United Kingdom; 5 University Department of Radiology, Addenbrooke’s Hospital, Cambridge, United Kingdom; Fred Hutchinson Cancer Research Center, UNITED STATES

## Abstract

**Background:**

The major clinical challenge in the treatment of high-grade serous ovarian cancer (HGSOC) is the development of progressive resistance to platinum-based chemotherapy. The objective of this study was to determine whether intra-tumour genetic heterogeneity resulting from clonal evolution and the emergence of subclonal tumour populations in HGSOC was associated with the development of resistant disease.

**Methods and Findings:**

Evolutionary inference and phylogenetic quantification of heterogeneity was performed using the MEDICC algorithm on high-resolution whole genome copy number profiles and selected genome-wide sequencing of 135 spatially and temporally separated samples from 14 patients with HGSOC who received platinum-based chemotherapy. Samples were obtained from the clinical CTCR-OV03/04 studies, and patients were enrolled between 20 July 2007 and 22 October 2009. Median follow-up of the cohort was 31 mo (interquartile range 22–46 mo), censored after 26 October 2013. Outcome measures were overall survival (OS) and progression-free survival (PFS). There were marked differences in the degree of clonal expansion (CE) between patients (median 0.74, interquartile range 0.66–1.15), and dichotimization by median CE showed worse survival in CE-high cases (PFS 12.7 versus 10.1 mo, *p* = 0.009; OS 42.6 versus 23.5 mo, *p* = 0.003). Bootstrap analysis with resampling showed that the 95% confidence intervals for the hazard ratios for PFS and OS in the CE-high group were greater than 1.0. These data support a relationship between heterogeneity and survival but do not precisely determine its effect size. Relapsed tissue was available for two patients in the CE-high group, and phylogenetic analysis showed that the prevalent clonal population at clinical recurrence arose from early divergence events. A subclonal population marked by a *NF1* deletion showed a progressive increase in tumour allele fraction during chemotherapy.

**Conclusions:**

This study demonstrates that quantitative measures of intra-tumour heterogeneity may have predictive value for survival after chemotherapy treatment in HGSOC. Subclonal tumour populations are present in pre-treatment biopsies in HGSOC and can undergo expansion during chemotherapy, causing clinical relapse.

## Abstract 2

In this study, James Brenton and colleagues demonstrate that quantitative measures of intratumoural heterogeneity may have predictive value for survival after chemotherapy treatment in high-grade serous ovarian cancer.

## Editors’ Summary

**Background:**

Every year, nearly 250,000 women develop ovarian cancer, and about 150,000 die from the disease. Ovarian cancer occurs when a cell on the surface of the ovaries (two small organs in the pelvis that produce eggs) or in the Fallopian tubes (which connect the ovaries to the womb) acquires genetic changes (mutations) that allow it to grow uncontrollably and to spread around the body (metastasize). For women whose ovarian cancer is diagnosed when it is confined to its site of origin, the outlook is good. About 90% of these women survive for at least five years. However, ovarian cancer is rarely diagnosed this early. Usually, by the time the cancer causes symptoms (often only vague abdominal pains and mild digestive disturbances), it has spread into the peritoneal cavity (the space around the gut, stomach, and liver) or has metastasized to distant organs. Patients with advanced ovarian cancer are treated with a combination of surgery and platinum-based chemotherapy, but only a quarter of such women are still alive five years after diagnosis, and the overall five-year survival rate for ovarian cancer is less than 50%.

**Why Was This Study Done?:**

The major clinical challenge in the treatment of high-grade serous ovarian cancer (HGSOC; the most common type of ovarian cancer) is the development of resistance to platinum-based chemotherapy. If we knew how this resistance develops, it might be possible to improve the treatment of HGSOC. Tumors are thought to arise from a single mutated cell that accumulates additional mutations as it grows and divides. This process results in the formation of subpopulations of tumor cells, each with a different set of mutations. Experts think that this “intra-tumor heterogeneity” gives rise to tumor subclones that possess an evolutionary advantage over other subclones (they might, for example, grow faster or be resistant to chemotherapy) and that eventually dominate the tumor (“clonal expansion”). Here, the researchers investigate whether clonal evolution and the emergence of subclonal tumor populations explains the development of chemotherapy-resistant HGSOC by undertaking evolutionary inference and phylogenetic quantification of the heterogeneity of samples taken from women with HGSOC at different times and from different places in their body. Evolutionary inference and phylogenetic quantification are analytical approaches that can be used to reconstruct the evolutionary history (“family tree”) of a tumor.

**What Did the Researchers Do and Find?:**

The researchers used an algorithm (a step-by-step procedure for data processing) called MEDICC to analyze detailed genetic data obtained from 135 spatially and temporally separated samples taken from 14 patients with HGSOC who had received platinum-based chemotherapy. The researchers report that there were marked differences in the degree of clonal expansion among the patients. When they split the patients into two groups based on the degree of clonal expansion in their tumors (CE-high and CE-low), patients with tumors classified as CE-high had a shorter progression-free survival time than patients with tumors classified as CE-high (10.1 months compared to 12.7 months) and a shorter overall survival time (23.5 months compared to 42.6 months). Moreover, a type of statistical analysis called bootstrap analysis, which tests for the robustness of the result, indicated that having CE-high tumors was likely to increase a patient’s risk of a poor outcome. Finally, phylogenetic analysis of samples taken from two patients before and after relapse and analysis of a *NF1* deletion (*NF1* encodes neurofibromin 1, a tumor suppressor protein that prevents uncontrolled cell growth; *NF1* is frequently mutated in HGSOC) indicated that a resistant subclonal population was already present in the patients’ tumors before treatment began.

**What Do These Findings Mean?:**

These findings show that clonal expansion occurs between diagnosis and relapse in HGSOC, that there are marked differences in the degree of clonal expansion among patients, and that a high degree of clonal expansion may have a negative effect on survival. The accuracy of these findings is limited by the small number of patients included in the study, and it is likely that the analyses reported here overestimate the effect of clonal expansion on patient outcomes. Nevertheless, the researchers suggest that, provided larger patient studies yield similar results, quantitative measures of intra-tumor heterogeneity might be useful as patient-specific prognostic markers in HGSOC. That is, measures of intra-tumor heterogeneity might eventually help clinicians to predict which of their patients with ovarian cancer are likely to have the best outcomes after platinum-based chemotherapy.

**Additional Information:**

Please access these websites via the online version of this summary at http://dx.doi.org/10.1371/journal.pmed.1001789.

- The US National Cancer Institute provides information about cancer and how it develops (in English and Spanish), including detailed information about ovarian cancer
- Cancer Research UK, a not-for-profit organization, provides general information about cancer and how it develops, and detailed information about ovarian cancer
- The UK National Health Service Choices website has information and personal stories about ovarian cancer
- The not-for-profit organization Healthtalk.org provides personal stories about dealing with ovarian cancer; Eyes on the Prize, an online support group for women who have had cancers of the female reproductive system, also includes personal stories; the not-for-profit organization Ovarian Cancer Action also provides information, support, and personal stories about ovarian cancer
- Wikipedia provides information about clonal evolution in cancer, tumor heterogeneity, and phylogenetics (note that Wikipedia is a free online encyclopedia that anyone can edit; available in several languages)
- More information about the MEDICC algorithm is available

## Introduction

Intra-tumour genetic heterogeneity in cancer has been investigated for almost half a century [1,2], and recent advances in genomic technology have demonstrated diverse genetic changes within a single epithelial cancer [3–14]. Multiple sampling of primary and metastatic sites in breast, pancreas, and renal carcinoma has catalogued genetic divergence and shown that metastases from the same site can show organ-specific phylogenetic branches [5–8,12]. Deep sequencing of epithelial tumours has revealed the clonal compositions of individual clinical samples and has shown how major and minor subclones may co-exist [5,7,8,12,14,15]. These data extend earlier observations showing that there is significant intra-tumour heterogeneity in solid tumours, and suggest that tumours with sufficient heterogeneity may be able to explore the fitness landscape widely enough during selection pressure from chemotherapy to repopulate with a resistant subclone [16,17]. Although this phenomenon has been extensively demonstrated in haematological cancers [18,19], the sequence of clonal expansions (CEs) has not been comprehensively described in epithelial tumours or correlated with clinical outcome.

High-grade serous ovarian cancer (HGSOC) is genomically characterised by a ubiquitous *TP53* mutation, high-frequency somatic copy number alterations (CNAs), and whole genome duplications [20–22]. Oncogenic mutations are rare, and most nonsynonymous changes are seen in tumour suppressor genes, including somatic mutations in *TP53*, *BRCA1*, *BRCA2*, *RB1*, and *NF1* [22]. Loss of *NF1*, an inhibitor of RAS signalling, may occur by point mutation or structural rearrangement and may be present in subclonal populations [23–27]. The typical clinical presentation of HGSOC is with extensive abdominal disease, involving multiple implantation sites throughout the abdomen. Intra-tumour heterogeneity may contribute to acquired resistance in HGSOC [3,4,28–30], but quantitation of the degree of heterogeneity and its relationship to changes in the course of treatment or the development of resistance is unknown.

Accurately reconstructing the evolutionary history of cancer cells in an unbiased manner improves the quantification of tumour heterogeneity. However, inferring phylogenetic trees in cancers that have highly frequent somatic CNAs is particularly difficult because of the unknown phasing of parental alleles and the horizontal dependencies between adjacent genomic loci. Previous work has used ad hoc thresholds or visual analysis [15,31]. We recently developed the Minimum Event Distance for Intra-tumour Copy Number Comparisons (MEDICC) algorithm, which provides accurate estimates of evolutionary distances between tumour samples by determining the optimum phasing of major and minor alleles from copy number or whole genome sequencing (WGS) data [32]. A numerical measure of the degree of heterogeneity can also be derived by transforming the pairwise minimum event distances [32].

To address the hypothesis that quantitative measures of intra-tumour heterogeneity could predict outcome in HGSOC, we collected multiple spatially and temporally separated tumour samples from 14 women undergoing chemotherapy for HGSOC, and used formal methods to reconstruct the evolutionary history of the disease within each patient from whole genome copy number profiles.

## Materials and Methods

### Ethical Consent

Tissue samples were obtained from the prospective CTCR-OV03 and CTCR-OV04 clinical studies, which were designed to collect imaging, blood, and tissue samples for exploratory biomarker studies. All patients provided written, informed consent for participation in these studies and for the use of their donated tissue, blood specimens, and anonymized data for the laboratory studies carried out. The CTCR-OV03 and CTCR-OV04 studies were approved by the Suffolk Local Research Ethics Committee (reference 05/Q0102/160) and Cambridgeshire Research Ethics Committee (reference 08/H0306/61).

### SNP Arrays

DNA extraction was performed using the DNeasy Blood & Tissue Kit (Qiagen) following the manufacturer’s instructions. In total, 177 samples from 18 patients were profiled for copy number aberrations using Affymetrix Genome-Wide SNP 6.0 arrays (S1 Table). Hybridisation of DNA to SNP 6.0 arrays was performed by AROS Applied Biotechnology following the manufacturer’s protocol. Array data are available online at the NCBI Gene Expression Omnibus under accession number GSE40546. The datasets were segmented using PICNIC [33] (using the “primary” option), which further corrects for cellularity and estimates integer major and minor copy numbers.

### Evolutionary Inference and Tree Robustness

The MEDICC algorithm and methods for copy number reconstruction and quantification of heterogeneity have been described previously [32]. We determined the CE and temporal heterogeneity (TH) indices as described for patients with more than three samples and where paired biopsy and surgery samples were available.

### Paired-End Sequencing and Breakpoint Validation

DNA extracted from tumour samples and from matched normal blood was processed using the Illumina Paired-End Sample Prep Kit. Paired-end WGS (41 bp; in some cases 50 bp trimmed to 41 bp) was performed on the Illumina Genome Analyzer IIx, where the median number of read pairs for each library was 153 million and the median sequencing depth was ×16.7. Sequencing data were processed using analysis pipelines as previously described [34]. Briefly, reads were aligned using BWA [35] and Novoalign (Novocraft Technologies), and discordantly mapped read pairs were used to identify putative structural variants using a custom pipeline. PCR primers for validating structural variants were designed using Primer3 [36].

Deletion and insertion breakpoints from WGS were considered confirmed if there was >50% reciprocal overlap of copy number decrease or increase in the SNP array data. Additionally, deletion, insertion, inter-chromosomal, and inversion breakpoints were considered confirmed if both ends of a breakpoint were within 10 kb of copy number breakpoints in any of the sequenced samples of the tumour.

### Mutation Detection by Resequencing

The coding sequences of *TP53*, *BRCA1*, *BRCA2*, *PTEN*, *PIK3CA*, *EGFR*, and *APC* were sequenced using the TAm-Seq method using the Fluidigm Access Array 48.48 platform as described previously [37] with minor modifications: pre-amplification steps were omitted, as high-molecular-weight DNA was extracted from fresh-frozen tumour specimens, and 50 ng of sample DNA and multiplexed primers was used in the target-specific amplification step. Primer sequences are available upon request. Sequencing data analysis and variant verification was performed using an in-house-developed pipeline and IGV software as previously described [37,38].

### Digital PCR

Digital PCR was performed using the Fluidigm Biomark microfluidic system according to the manufacturer’s instructions. Primers were designed spanning the *NF1* deletion (forward: 5′-TTTTGTTTACGAGCACAGATAACC-3′; reverse: 5′-GAAACAGAAGATGACAGCAAAGAA-3′). Reaction mixes were prepared containing 1× TaqMan Gene Expression Master Mix (Applied Biosystems), 1× EvaGreen DNA binding dye (Biotium), 1× DNA Binding Dye Sample Loading Reagent (Fluidigm), and 10 nM primers and template DNA. Prior to loading into a 12.765 Fluidigm digital chip, reactions were heated to 95°C for 1 min and placed on ice. Reactions were thermocycled at 50°C for 2 min, 95°C for 10 min, followed by 55 cycles of 95°C for 15 s and 56°C or 60°C for 1 min. Digital PCR was also performed on the same samples using an assay for the p.R175H mutation in *TP53* as previously described [39] (forward: 5′-CCATCTACAAGCAGTCAC-3′; reverse: 5′-GTCACCATCGCTATCTGAG-3′; mutant-specific probe: [6FAM]-TTGTGAGGCACTGCCCCC-[BHQ1]; wild-type-specific probe: [HEX]-TTGTGAG-GCGCTGCCCCC-[BHQ1]). The proportion of tumour cells with the *NF1* deletion was calculated from the estimated counts of the assays for both the *NF1* deletion and mutant *TP53* p.R175H.

### Study Design

Cases were retrospectively selected from available tumour samples from the CTCR-OV03 and CTCR-OV04 studies. The CTCR-OV03 study has been previously described [40] and was a prospective, single-institution, protocol-driven study with eligibility criteria of (a) clinical diagnosis of advanced ovarian cancer (International Federation of Gynecology and Obstetrics stage 3 or higher), (b) gynaecology-oncology multidisciplinary team recommendation for neoadjuvant chemotherapy treatment before interval debulking surgery, (c) measurable disease at staging based on computed tomography of the abdomen and pelvis, and (d) no contraindications to MRI [40]. Samples were stored in RNAlater (Life Technologies) immediately after acquisition and later histologically examined and scored for cellularity by a specialist gynaecological pathologist (M J.-L.). When selecting cases for analysis, 14/28 CTCR-OV03 patients were excluded from the planned analysis (six had tumours that were not HGSOC histology, five had no research tissue available, and three had samples from only one time point or one metastatic site), leaving 14 for genomic profiling. Four additional “convenience” cases were obtained from the CTCR-OV04 study and were selected by availability of ≥3 spatially discrete, fresh-frozen tumour biopsies. All patients were enrolled between 20 July 2007 and 22 October 2009. The follow-up was censored after 26 October 2013, with median duration of 31 mo (interquartile range [IQR] 22–46 mo; range 7–53 mo). Progression-free survival (PFS) was defined as the interval between the date of the original pathology report confirming ovarian cancer and the date of progression measured by RECIST 1.1, Gynecologic Cancer Intergroup CA 125 criteria, or symptomatic progression. Overall survival (OS) was defined as the interval between the date of the original pathology report confirming ovarian cancer and the date of death from any cause. Clinical details, including CA 125 measurements, stage, and residual disease after debulking surgery, were abstracted from clinical records by research staff.

### Statistical Analysis Methods

A detailed transcript of all statistical analyses using R and Sweave is provided in S1 Protocol.

## Results

**Fig 1:**
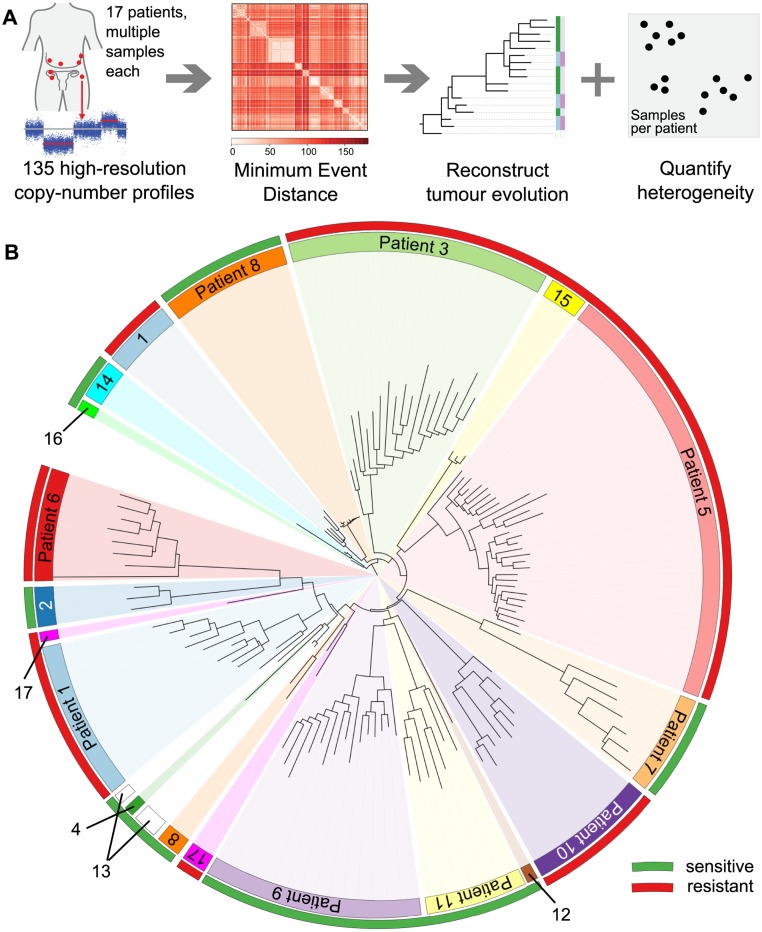
Overview of the analysis and the clinical dataset. (A) Numerical quantification of intra-tumour genetic heterogeneity by evolutionary comparisons. Copy number profiles from 135 metastatic sites were obtained for 17 patients with HGSOC. The MEDICC algorithm was used to compute minimum event distances between profiles and to reconstruct the evolutionary history for each sample, enabling numerical quantification of both spatial and TH and CE for each patient. (B) HGSOC exhibits significant patient-specific intra-tumour genetic heterogeneity. Neighbour-joining tree of all samples based on total copy number. Samples from each patient (coloured inner circular bar) cluster into clades. The outer circular bar indicates HGSOC classified as resistant versus sensitive to treatment based on survival: red, resistant, PFS < 12 mo; green, sensitive, PFS > 12 mo. No immediate clustering of patients into sensitive and resistant subgroups is visible.

We collected 177 temporally and spatially distinct HGSOC samples from 18 patients undergoing platinum-based chemotherapy (Fig. 1A). Copy number profiles were obtained with Affymetrix Genome-Wide SNP6.0 arrays (Table 1) and segmented using PICNIC [33]. Of the 18 patients, 17 had neoadjuvant chemotherapy (Table 1). The median number of chemotherapy cycles prior to interval debulking surgery was three (range 3–7). One patient had primary surgery followed by adjuvant chemotherapy. Data from 39/177 arrays were excluded after profiling because of tumour cellularity < 50% or high noise, resulting in removal of one patient from the analysis (6/6 samples excluded), leading to a final total of 17 patients included in the following analyses. In all, 31/39 excluded arrays were from samples obtained from interval debulking surgery following pre-operative chemotherapy (S1 Table). Analyses of clonal evolution were performed using whole genome bi-allelic integer copy number profiles of 135 tumour samples from 14 patients who had ≥3 samples. Ten patients had samples both from biopsy prior to chemotherapy treatment and from interval debulking surgery, allowing for comparison of temporal effects. Two patients had relapsed ascites samples (S1 Table).

**Table 1:** Summary of samples from the CTCR-OV03/04 clinical studies.

Patient Number	Patient Age^a^	Tumour Stage	Response^b^	Treatment	CA 125 Reduction^c^	PFS	OS	Number of Samples^d^	TH Index	CE Index	*p-*Value for Star Topology^e^
1	I	IV	PR	Neo	−93	271	511	16/20	4.73	1.26	<0.001
2	IV	IV	PR	Neo	−92	363	977	3/5	NA	0.71	0.67
3	V	IV	SD	Neo	−92	153	209	18/20	3.74	1.24	<0.001
4	I	IIIC	PR	Neo	−91	616	625	1/3	NA	NA	NA
5	IV	IV	PR	Neo	−76	303	547	29/29	3.8	1.47	<0.001
6	III	IV	SD	Neo	−80	298	744	8/8	6.59	0.73	0.001
7	IV	IV	PR	Neo	−43	358	1,587	7/8	3	0.68	<0.001
8	II	IIIC	PR	Neo	−24	373	889	11/14	3.42	2.24	<0.001
9	VI	IV	SD	Neo	−100	563	1,278	15/16	4.49	0.65	<0.001
10	III	IIIC	PR	Neo	−87	303	1,139	9/11	4.72	0.87	<0.001
11	III	IIIC	PR	Neo	−98	382	1,556	7/17	5.7	0.48	0.28
12	III	IIIC	SD	Neo	−88	534	1,565	1/4	NA	NA	NA
13	III	IIIC	PR	Neo	−28	776	1,166	3/3	NA	0.62	0.48
14	IV	IIIC	PR	Neo	NA	601	1,513	3/4	4.62	0.61	0.74
15	II	IV	SD	Neo	NA	332	706	3/5	NA	0.74	0.74
16	III	IIIC	PR	Neo	NA	1,380	1,405	1/3	NA	NA	NA
17	III	IIIC	SD^f^	PS	NA	293	849	3/4	NA	0.86	0.64

The table shows patients identified for study and the number of samples available before and after quality control. Patients with <3 samples could not be evaluated for TH and CE indices. Patients with CE index but no TH index did not have paired pre-chemotherapy biopsy and interval debulking surgery samples available.

^a^Patient age was segmented into brackets as follows: I, 45–50 y; II, 46–55 y; III, 56–60 y; IV, 61–65 y; V, 66–70 y; VI, 71–75 y.

^b^Response according to RECIST evaluation: PD, progressive disease; PR, partial response; SD, stable disease.

^c^CA 125 tumour marker reduction (percentage),

^d^Number of samples used for analysis (out of all samples taken in study).

^e^Test for star topology (BH corrected).

^f^Not comparable to other SD cases as treatment modalities were different.

NA, not available; Neo, neoadjuvant; PS, primary surgery.

To exclude potential confounding effects on heterogeneity, we performed a detailed pathology review of all paraffin blocks from each patient. No significant differences in morphology or growth pattern were observed between metastatic sites in any patient (S1 Fig). In addition, we performed tagged-amplicon resequencing of tumour tissue for genes commonly somatically mutated in HGSOC. *BRCA1* and *BRCA2* were also included in the sequencing panel as OS is significantly improved in women with germ line mutations. In all, 15/17 patients had a mutation in *TP53* consistent with HGSOC (Table 2) [20]. Of the two wild-type *TP53* cases, patient 12 had strong nuclear p53 protein accumulation consistent with p53 dysfunction, and patient 3 was reclassified as a synchronous HGSOC and high-grade uterine serous papillary carcinoma (based on simultaneous invasive uterine and ovarian lesions together with positive WT1 immunohistochemistry). No germ line mutations in *BRCA1* and *BRCA2* were identified. Patient 14 had a nonsense mutation in *BRCA2*, and patient 2 showed variants of uncertain significance in *BRCA1* somatic nonsense mutation in *BRCA2*, and patient 2 showed variants of uncertain significance in *BRCA1* and *BRCA2* (Table 2). We examined the copy number profiles for evidence of specific mutator phenotypes that would alter the propensity for evolutionary change, but did not find any patient with features of the tandem duplicator phenotype [34,41].

**Table 2:** Mutations detected in samples from CTCR-OV03 and CTCR-OV04 patients using TAm-Seq.

Patient Number	Effect	Gene	Protein Change	cDNA Change	RefSeq ID
1	MS	*TP53*	p.Y234C	c.A701G	NM_000546
2	NS	*TP53*	p.Y234X	c.C702A	NM_000546
2	MS	*BRCA1*	p.Y179C	c.A536G	NM_007294
2	MS	*BRCA1*	p.N550H	c.A1648C	NM_007294
2	MS	*BRCA1*	p.F486L	c.T1456C	NM_007294
2	MS	*BRCA2*	p.E1110V	c.A3329T	NM_000059
3	ND	*TP53*			
4	MS	*TP53*	p.H214R	c.A641G	NM_000546
5	MS	*TP53*	p.C141R	c.T421C	NM_000546
6	FS	*TP53*	p.P153fs	c.459_469del11	NM_000546
7	MS	*TP53*	p.R273C	c.C817T	NM_000546
7	MS	*APC*	p.S2596A	c.T7786G	NM_000038
8	MS	*TP53*	p.R175H	c.G524A	NM_000546
8	Silent	*BRCA2*	p.G1552G	c.T4656C	NM_000059
9	FS	*TP53*	p.I195fs	c.583_584dupA	NM_000546
10	MS	*TP53*	p.S215G	c.A643G	NM_000546
10	MS	*APC*	p.D1714N	c.G5140A	NM_000038
11	NS	*TP53*	p.R306X	c.C916T	NM_000546
12	ND	*TP53*			
13	MS	*TP53*	p.Y236S	c.A707C	NM_000546
13	Silent	*BRCA2*	p.V465V	c.A1395C	NM_000059
14	MS	*TP53*	p.V216L	c.G646T	NM_000546
14	NS	*BRCA2*	p.L2732X*	c.T8195A	NM_000059
15	MS	*TP53*	p.C135R	c.T403C	NM_000546
16	MS	*TP53*	p.C275Y	c.G824A	NM_000546
17	MS	*TP53*	p.R273H	c.G818A	NM_000546

Patient 14 had a deleterious somatic nonsense mutation (p.L2732X*) in *BRCA2*. This mutation was not detected in two independent germ line DNA samples from patient 14. All other *BRCA1/2* mutations were not pathogenic or were of no/unknown clinical importance according to the Breast Cancer Information Core Database and the LOVD-IARC database.

FS, frameshift; MS, missense; ND, no mutation detected; NS, nonsense; silent, silent mutation.

We recently developed the MEDICC package [32] to reconstruct patient-specific evolutionary trees and quantify heterogeneity in tumour samples using methods that employ a minimum evolution criterion to measure the genetic divergence between copy number profiles. This algorithm estimates evolutionary distances between samples based on the minimum number of segmental amplifications and deletions needed to transform one genomic profile into another using optimised allele-specific assignment of major and minor copy numbers. Using MEDICC we reconstructed evolutionary trees for 14 patients with three or more samples (Fig. 1A). Circos plots and evolutionary trees for all patients can be found in S3–S16 Figs.

### Patient-Specific Copy Number Changes Cluster by Anatomical Site

As expected, CNAs were highly patient-specific. A tree reconstructed from applying MEDICC to all 135 samples grouped samples by patient (Fig. 1B). However, no clustering into subgroups with HGSOC resistant or sensitive to a second line of treatment was evident by this analysis (outer colour bar, Fig. 1B). We next applied MEDICC to each patient individually. Copy number changes within each patient showed strong divergence overall, with a median of 54.5 (IQR 32.5–65.6) genomic events. Divergence from the hypothetical normal genome was, as expected, larger, with a median of 104 events (IQR 57.8–112). Three patients showed less marked changes; reconstruction of events for patients 1 and 8 had limited phylogenetic signal owing to low divergence or limited numbers of samples, and in patient 1 we found very high heterogeneity and strong CE (discussed below).

**Fig 2:**
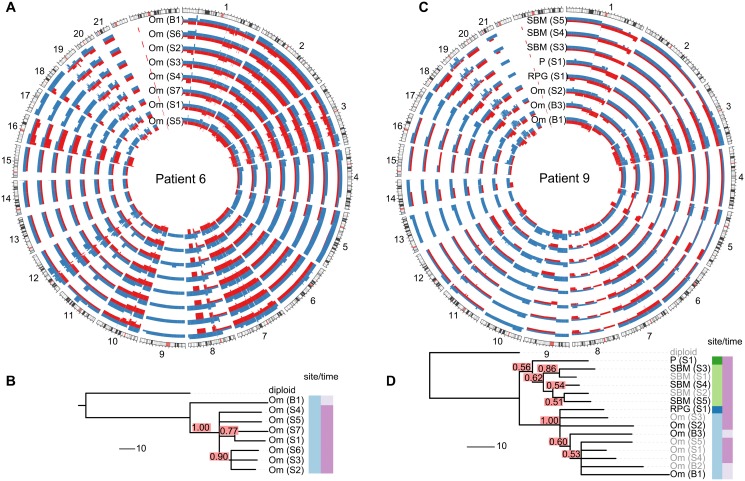
Examples of spatial and temporal heterogeneity in HGSOC. (A and C) Total copy number profiles show strong overall conservation. As examples, a representative subset of the allele-specific genomic copy number profiles of patients 6 and 9 are shown. Separate alleles are indicated in red and blue. (B and D) Genomic changes between biopsy and surgery reveal tumour evolution. The black sample names in the trees indicate the samples shown in the Circos plots. Confidence values for each split are printed in red boxes. The colour-coded bars on the right of the phylogenies indicate different sites (left column) and different sampling times (right column). Branch lengths indicate number of genetic events as determined by MEDICC (scale bar shows ten events). Om, omentum; P, peritoneum; RPG, right paracolic gutter; SBM, small bowel mesentery.

Metastasis of HGSOC is thought to occur by physical shedding from the invasive lesions in the fallopian tube onto pelvic structures and into the abdomen, where physiological recirculation of ascites fluid facilitates widespread seeding of tumour cells. Evolutionary clades in the patient-specific trees often agreed with the anatomical sites where the sample was taken (see Fig. 2 and individual trees in S3–S16 Figs.). For example, patient 9 showed clear separation of omentum and small bowel mesentery samples (Fig. 2D), with right paracolic gutter and peritoneum as the respective out-groups. Genetic markers of this divergent evolution included Chromosomes 2q and 3p as well as amplifications on Chromosome 10 (Fig. 2C). Higher resolution analyses with paired-end WGS on samples from six patients confirmed additional divergent genetic change at higher resolution. For example, in patient 9, there were three deletion breakpoints and an insertion breakpoint that were present only in the omentum sample, and three deletion breakpoints that were only in the peritoneal sample (S18 Fig).

### Analysis of Tree Topologies Suggests Metastasis-to-Metastasis Spread with Changing Evolutionary Rates

**Fig 3:**
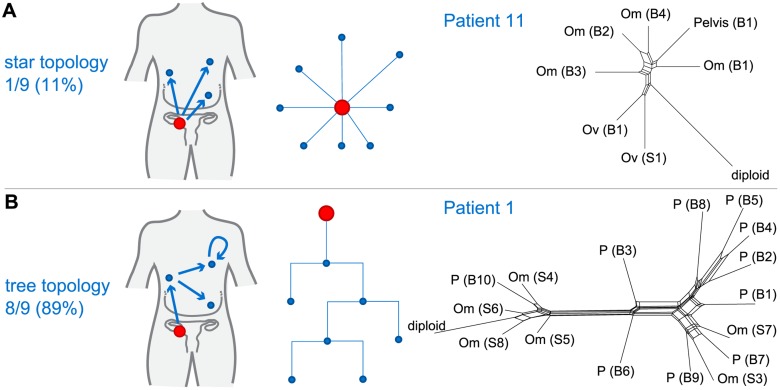
Branching patterns in HGSOC. (A) Radial pattern of metastatic spread leads to a star topology. The schematic shows how the evolutionary relationships are predicted to have a star-like topology if all metastases (blue) are derived from the primary lesion (red). A neighbour-net representation of the evolutionary distances from patient 11 shows deviation from a tree structure (right). (B) Branched metastatic spread leads to a tree topology. The schematic shows that evolutionary history is predicted to be tree-like if metastases create new metastases (including metastasis-to-metastasis spread). A neighbour-net representation of the distance matrix for patient 1 shows a tree-like structure (right). The number and proportion of patients classified to star or tree topology are shown. Labels on trees indicate site of metastasis (Om, omentum; Ov, ovary; P, peritoneum). Sample identifiers indicate whether the sample was collected from pre-chemotherapy biopsy (B) or interval debulking surgery (S).

We next considered whether the observed spatial heterogeneity arose from metastasis-to-metastasis spread or by successive metastases from the primary cancer site by examining the pattern of the evolutionary relationships between metastatic samples within each patient. Given a fixed number of metastases, two scenarios are possible: if only the primary tumour gives rise to metastatic clones, the resulting evolutionary tree will have a star topology (Fig. 3A). By contrast, if cells retain their metastatic potential after metastasis, ongoing spread and associated genetic change will lead to a fully branched evolutionary tree (Fig. 3B).

We used MEDICC to test the null hypothesis for each patient that the evolutionary distances were derived from a star topology [32]. To verify and visualise the findings we applied the neighbour-net method [42], which captures non-tree-like evolutionary signals in distance data. From nine patients with ≥3 samples, eight showed significant branching (*p* < 0.05, chi-squared test for goodness of fit with Benjamini and Hochberg correction for false discovery rate), supporting the model of metastasis-to-metastasis spread. Patient 11 showed only a weak tree structure, and the null hypothesis could not be rejected (*p* = 0.22; Fig. 3A).

Next we compared evolutionary distances within each patient to investigate whether evolutionary change occurs at a constant rate (clock-like evolution). After correction for multiple testing, two out of 14 patients (14.3%) showed significant non-clock-like evolutionary trajectories (*p* < 0.05). We conclude that HGSOC shows metastasis-to-metastasis spread and that heterogeneity is generated through ongoing clonal evolution with potentially unknown mutator phenotypes present.

### Small Changes in Heterogeneity Occur during Neoadjuvant Therapy

As most metastases are established before onset of treatment, we next investigated the rate of ongoing clonal evolution by examining samples before and after neoadjuvant chemotherapy. The average genomic change during treatment (TH index) was quantified using MEDICC. To ensure that differences were not due to cellularity, we compared histopathology estimates between the pre-chemotherapy biopsies and surgical specimens and found no significant differences (*t*-test, *p* = 0.7). MEDICC measures TH by mapping genomes into a high-dimensional space, termed the “mutational landscape” [16,17], in which distances correspond to evolutionary distances between genomes. The TH index is then calculated as the distance between the robust centres of mass of the biopsy and surgery samples [32], leading to a robust estimate of change during treatment. Visual analysis of Circos plots showed strong overall conservation, indicating that the main karyotypes for each cancer were established before onset of treatment. Quantitative analysis with MEDICC detected genomic differences between biopsy and surgery samples, showing on average 46 (standard deviation 13) new genomic events (Figs. 2A, 2C, and S3–S16). For example, for patient 6 there was a profound difference between the two sample subgroups (TH index 0.66), with early and long branching of the pre-treatment omental biopsy sample away from the remaining omental samples, indicating divergent evolution (Fig. 2A and 2B). The copy number events responsible for this divergence included deletions on Chromosomes 1p, 1q, 3p, 7q, 9q, and 11p. In patient 9, one of the three omentum samples differed in 18.1% of its genome from the omentum samples at surgery (Fig. 2C and 2D). We concluded that HGSOC shows detectable changes during neoadjuvant chemotherapy (median 75 d), but these are minor compared to the overall changes from the onset of the disease.

### HGSOC is Frequently Polygenomic and Shows Variable Clonal Expansion

It has previously been shown in breast cancer that CEs of minor subpopulations of cells lead to polygenomic tumours, while other tumours appear monogenomic [6]. These CEs are potentially modulated by selection pressure from chemotherapy (or other factors) and might have prognostic value. Using MEDICC allowed statistical quantification of the degree of CE on a continuous scale (CE index) by testing for local spatial clustering of genomes in the mutational landscape [32].

**Fig 4:**
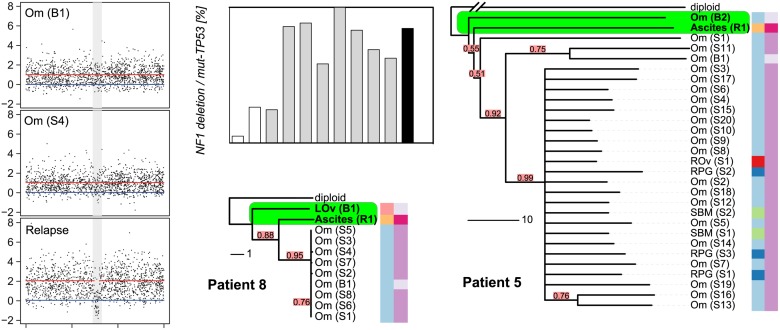
Relapse is an early diverged clonal expansion of a low-prevalence subclone of pre-treatment disease. Array copy number profiles (left) from patient 8 detected a focal *NF1* deletion in the relapsed ascites sample that was not observed in the pre-chemotherapy or interval debulking samples. The bar plot shows the results of digital PCR for the *NF1* breakpoint from pre-chemotherapy (white bars), interval debulking (grey bars), and relapsed ascites (black) samples. Phylogenetic trees for patients 8 and 5 are shown. The relapsed clonal population for each case is placed next to the pre-chemotherapy biopsy sample, indicating early branching events from the diploid. The length of each branch indicates the degree of divergence. Colour coding and sample identifiers are as for Fig. 3. LOv, left ovary; Om, omentum; SBM, small bowel mesentery; RPG, right paracolic gutter.

We found the CE index to be variable across the cohort (median 0.73, IQR 0.65–1.24). As there was no clinically defined cutoff point for CE, the median value was used to divide patents into two groups (CE-low versus CE-high). Patients with in the CE-low group, for example patient 11, showed linear emission of samples throughout the tree and had homogenous branch lengths (S12 Fig). By contrast, patients 5 and 8, in the CE-high group, showed marked CE (CE index 1.47 and 2.24, respectively), with multiple samples in strongly diverging subclades (Fig. 4).

### Patients with Tumours with High Clonal Expansion Show Short Survival and Resistant Relapse

It has been proposed that for a tumour to overcome the selection pressure applied by chemotherapy, it needs to be able to efficiently explore the mutational landscape [17]. Therefore, we hypothesized that polygenomic tumours that have already undergone CEs are likely to be at an advantage for acquiring other mutations for survival during treatment.

**Fig 5:**
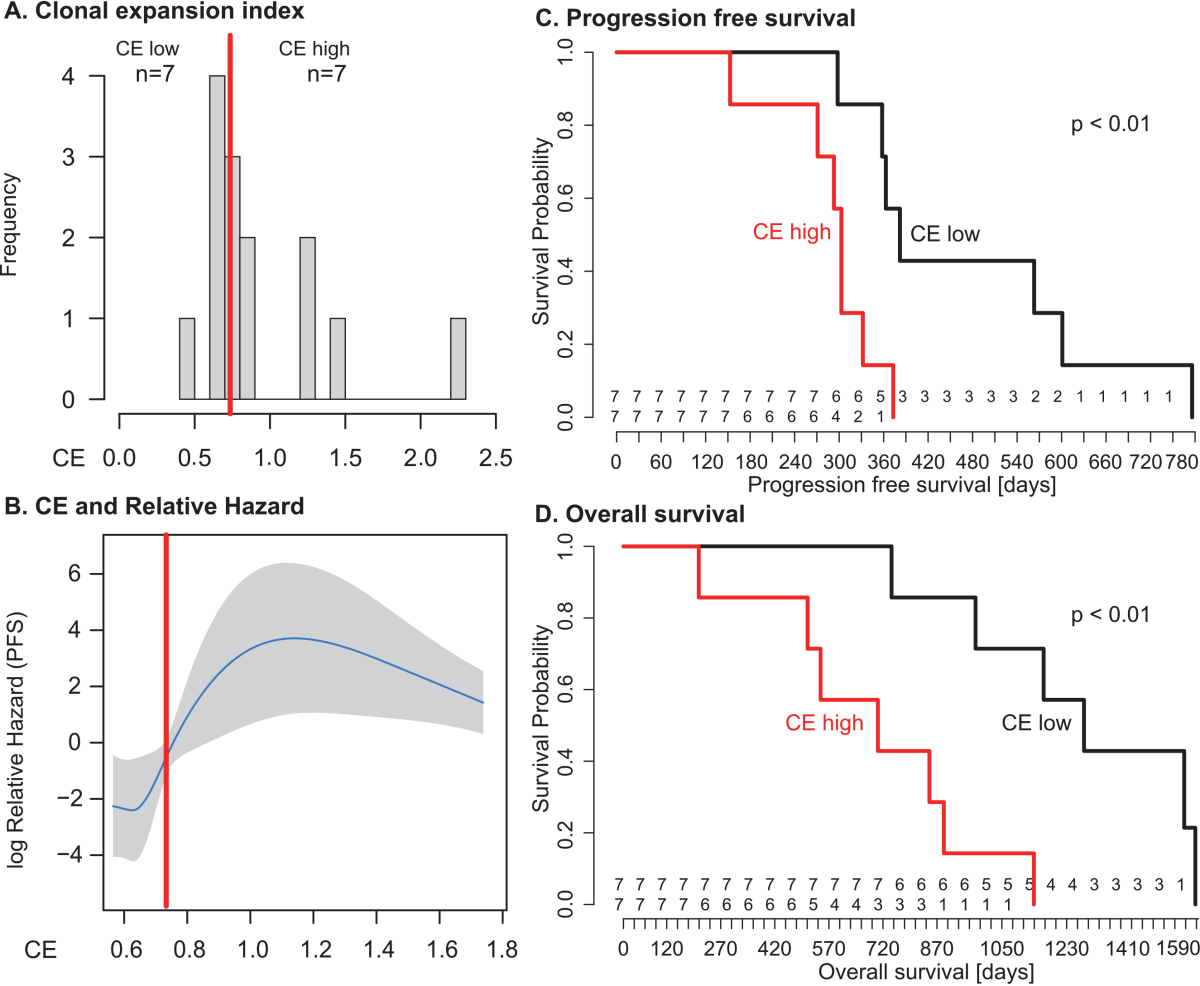
Clonal expansion index stratifies patients into prognostic subgroups. (A) Distribution of CE index over all patients and the respective group sample sizes (*n*). The red line indicates median CE = 0.73, dichotomizing the cases into equal-sized CE-low and CE-high groups. (B) The relationship between CE and relative hazard is nonlinear. The fit line is generated from the multivariable model incorporating penalised spline smoothing. Grey shading indicates the 95% confidence interval for log hazard. Extreme CE values are not shown as the spline smoothing algorithm disregards values outside the 95% range. The median (red line) separates a region of low hazard from a region of high hazard indicated by non-overlapping confidence intervals. (C and D) The CE-low and CE-high groups show a statistically significant difference in PFS (log-rank *p* < 0.01) and OS (log-rank *p* < 0.01). Numbers at risk are given above the *x*-axis for the CE-low (top) and CE-high (bottom) groups.

We used the log-rank test to test for differences in PFS and OS between the CE-low and CE-high groups (Fig. 5). Survival was shorter in patients in the CE-high group (PFS 12.7 versus 10.1 mo, *p* = 0.009; OS 42.6 versus 23.5 mo, *p* = 0.003; Fig. 5). Being in the CE-high group was an independent predictor of survival in a multivariable Cox hazard regression analysis that included patient age, tumour stage, and residual disease after debulking surgery (PFS, *p* = 0.001; OS, *p* = 0.004). Survival differences were not significant between patients with low or high TH index (S1 Protocol).

We tested CE as a continuous variable in a Cox proportional hazard model, which assumes a linear relationship between CE and survival. In univariable analysis, the quantitative CE index had a borderline significant association with OS with hazard ratio (HR) = 2.7 (95% CI 0.96, 7.8; *p* = 0.06), but no significant association with PFS. In multivariable models that considered CE, patient age, tumour stage, and residual disease, CE as a quantitative variable was not significantly associated with OS or PFS (coefficient *p* = 0.64 and *p* = 0.76, respectively). We examined potential nonlinear effects of CE on survival using cubic spline methods. For PFS and OS, the relationship between CE and relative hazard was nonlinear, showing a step function effect with marked increase in hazard seen at CE values greater than 0.7–0.8 (Fig. 5B; S1 Protocol), similar to the median cut point. Given the small size of the patient cohort, we performed tests of robustness using bootstrap analysis with 10,000-fold resampling to test whether the HR for the CE-high group was likely to be greater than one (deleterious for outcome). The differences in PFS and OS remained significant (*p* < 0.05) in 82% and 92%, respectively, of the perturbed datasets. The bootstrapped derived median HR values for PFS and OS were HR = 7.1 (95% CI > 1) and HR = 11.4 (95% CI > 1), respectively.

### Resistant Subclonal Populations Are Present in Pre-Treatment Disease

The survival analyses suggested that the degree of CE could have effects on PFS and OS consistent with the hypothesis that cancers with high CE may have increased genetic diversity favouring the emergence of drug-resistant clones. We were able to explore this hypothesis in patients 5 and 8, who had additional samples collected at progression. Patient 8 had symptomatic progressive disease, with the development of ascites at 12 mo after completing chemotherapy. The phylogenetic reconstruction of her cancer showed early divergence of the ascites sample from the root (Fig. 4). Examination of the relapsed copy number profile revealed a new focal deletion at *NF1* that was not present in the pre-chemotherapy and interval debulking surgery samples (Fig. 4). *NF1* is recurrently mutated in HGSOC [22,23], which suggests this was unlikely to be a passenger event. As copy number profiles detect the dominant clone in a sample, we investigated the population structure of earlier samples using WGS to map the new *NF1* deletion, and digital PCR to accurately estimate the number of cells containing the *NF1* deletion in each sample. To prevent confounding effects from differences in tumour cellularity, the counts for the *NF1* deletion were expressed as a proportion of all mutant *TP53* counts. The *NF1* deletion was detected at 5% and 26% in the pre-treatment samples (absolute cellularity 80% and 41%) and in 25%–100% of the interval debulking samples (median cellularity 49%). Histological analysis of the left fallopian tube specimen removed at interval debulking confirmed a tubal primary site (S1 Fig). We therefore extended the digital PCR analysis to DNA from microdissected tissues from formalin-fixed tissue blocks including the left fallopian tube fimbria. The *NF1* deletion was present in 1.2% of the primary invasive carcinoma in the fallopian tube and 7.9% of the biopsy from the adjacent left ovarian metastasis (S2 Table).

In patient 5, inspection of the tree showed that the relapsed ascites sample also diverged early, with a long branch (Fig. 4) indicating marked divergent evolution. This divergence was associated with deletions on Chromosomes 1q, 15q, and 18q (S6A Fig). In summary, the *NF1* deletion, while part of the dominant subpopulation at relapse, was already present pre-treatment. As it is highly unlikely that this specific deletion arose twice independently in the course of tumour evolution, we conclude that the relapse was a CE of a minor subclone of pre-chemotherapy disease.

## Discussion

In this work, we tested the hypothesis that intra-tumour heterogeneity in HGSOC is correlated with survival. We also assessed whether certain subclonal populations contribute to treatment failure. Our experimental design combined two approaches: first, we analysed spatially and temporally separate tumour samples from 14 women undergoing chemotherapy. This approach estimates the genetic complexity of the cancer burden in an individual more accurately than sampling from a single location and time point [5,12,13,25]. Second, we applied formal methods (MEDICC) to infer the most parsimonious representation of genetic evolution in each patient’s cancer [32]. Importantly, our methods are fully unsupervised and are derived independently of the clinical data.

Our analyses showed marked differences in CE between patients and negative effects of high CE on survival. In two patients with very high CE, we demonstrated that clonal populations detected at relapse arose from early branching events, followed by divergent evolution and CE. Indeed, digital PCR of a *NF1* deletion that marked the predominant clonal population at relapse conclusively showed that this deletion was present at very low fraction in pre-treatment samples including the tubal primary site. We further showed that HGSOC generally evolves and spreads in a branching process with frequently changing rates of evolution. Taken together, these findings are consistent with previous data from cell-based studies and circulating tumour DNA assays that suggested that CE occurs between diagnosis and relapse in HGSOC [28,37].

Although the number of HGSOC samples studied here is relatively large compared to those of other publications, the size of the patient cohort prevents strong conclusions about effect sizes and clinical impact. We used a median value for CE as an unbiased cut point to avoid strong assumptions about the relationship between CE and survival, but given the limited sample size, it is likely that our analyses overestimate the prognostic effect of CE. It is notable that the majority of the samples that failed quality assurance (and were therefore excluded from estimations of heterogeneity) were taken after chemotherapy treatment, suggesting that these samples may have had better response to treatment [28]. This implies that the samples from which our heterogeneity measures were calculated may be enriched for more chemoresistant disease. We have not defined the minimum number of samples per case that are required for accurate estimates of CE, and this will require larger patient studies. Collecting these samples remains a major challenge for heterogeneity research, owing to the difficulties of collecting multiple fresh tissue samples at different treatment time points and the costs of detailed genomic profiling. Further technological development to use shallow WGS data from formalin-fixed, paraffin-embedded samples may be a useful approach to increasing statistical power in future studies.

Comparison of the effects of CE and TH on survival showed that TH was not predictive of PFS or OS. This was surprising, as we expected strong TH effects to be correlated with response, and therefore survival. There are several factors that may explain our finding. First, we were unable to take samples from the same tumour deposit before and after chemotherapy treatment. Therefore, apparent differences in TH could be confounded by spatial differences in tumour heterogeneity, rather than representing intrinsic changes in subclonal populations caused by chemotherapy treatment. Second, the time window for evolutionary changes to occur during chemotherapy was short compared to the genetic lifespan of each cancer. Third, both CE and TH showed moderate correlation with sample size (S1 Protocol). Sample size was not significant in a multivariable Cox model, but could potentially contribute to the predictive power of CE (S1 Protocol).

Our results are in disagreement with recent findings where the analysis of nonsynonymous mutations did not show effects of ongoing evolutionary change in HGSOC [43]. These findings were based upon exome sequencing of three patients, and the power of this assay for evolutionary inference is dependent upon the depth of sequencing achieved. It is also likely that the majority of nonsynonymous changes detected by exome sequencing are passenger or private mutations, which may explain why other studies have not found evidence of the strong evolutionary patterns that we see using CNAs.

Our phylogenetic reconstructions further allowed us to assess the robustness of the evolutionary trees, and thereby the certainty of placement of a sample in the tree. With this we addressed the question of when in the course of disease the relapse clone evolved. In both patients 5 and 8, we were able to determine an early branching point as the origin of relapse that shared an immediate ancestor with a pre-treatment sample. In larger datasets these methods could be applied to the identification of early driver events and may mitigate the difficulties of identifying therapeutically relevant CNAs in heterogeneous patients.

In summary, our approach has been to define the evolutionary trajectories of HGSOC using robust and accurate methods to reconstruct the phylogenetic trees for individual patients. This approach has the potential to act as a patient-specific prognostic indicator and may be a powerful tool to identify and calibrate surrogate genomic markers of CE.

## Supporting Information

S1 Data Data file for survival analysis. (ZIP)

S1 Fig Histopathology for case 8. (PDF)

S2 Fig Cross-patient loss of heterozygosity frequencies. (PDF)

S3 Fig Copy number profile and evolutionary tree for patient 1. Only selected copy number profiles are shown (A); these are marked in bold in the evolutionary tree (B). Individual alleles are coloured in red and blue. Confidence values for each split in the tree are given in red boxes. The colour bars to the right of the tree indicate different sampling sites (left) and sampling times (right). Branch lengths are given in number of rearrangement events. Particularly long branches are marked in green.
(PDF)

S4 Fig Copy number profile and evolutionary tree for patient 2. Caption as for S3 Fig.
(PDF)

S5 Fig Copy number profile and evolutionary tree for patient 3. Caption as for S3 Fig.
(PDF)

S6 Fig Copy number profile and evolutionary tree for patient 5. Caption as for S3 Fig.
(PDF)

S7 Fig Copy number profile and evolutionary tree for patient 6. Caption as for S3 Fig.
(PDF)

S8 Fig Copy number profile and evolutionary tree for patient 7. Caption as for S3 Fig.
(PDF)

S9 Fig Copy number profile and evolutionary tree for patient 8. Caption as for S3 Fig.
(PDF)

S10 Fig Copy number profile and evolutionary tree for patient 9. Caption as for S3 Fig.
(PDF)

S11 Fig Copy number profile and evolutionary tree for patient 10. Caption as for S3 Fig.
(PDF)

S12 Fig Copy number profile and evolutionary tree for patient 11. Caption as for S3 Fig.
(PDF)

S13 Fig Copy number profile and evolutionary tree for patient 13. Caption as for S3 Fig.
(PDF)

S14 Fig Copy number profile and evolutionary tree for patient 14. Caption as for S3 Fig.
(PDF)

S15 Fig Copy number profile and evolutionary tree for patient 15. Caption as for S3 Fig.
(PDF)

S16 Fig Copy number profile and evolutionary tree for patient 17. Caption as for S3 Fig.
(PDF)

S17 Fig Tree shapes and evolutionary patterns in resistant and sensitive cases. (PDF)

S18 Fig Paired-end sequencing of selected samples. (PDF)

S1 Protocol Sweave file for survival analysis. (RNW)

S1 Table Distribution of samples. (PDF)

S2 Table Digital PCR results. (PDF)

S3 Table Primers for digital PCR. (PDF)

S4 Table Copy number alteration feature selection results. (PDF)

S1 Text Case-by-case study of the CTCR-OV03/04 cohort. (PDF)

## Abbreviations

CE: clonal expansion
CNA: copy number alteration
HGSOC: high-grade serous ovarian cancer
HR: hazard ratio
IQR: interquartile range
MEDICC: Minimum Event Distance for Intra-tumour Copy Number Comparisons
OS: overall survival
PFS: progression-free survival
TH: temporal heterogeneity
WGS: whole genome sequencing
