# Supplementary material for: Spatial and Temporal Heterogeneity in High-Grade Serous Ovarian Cancer: A Phylogenetic Analysis
Source: PLoS Med. 2015 Feb 24;12(2):e1001789. doi: 10.1371/journal.pmed.1001789 (PMC4339382; doi:10.1371/journal.pmed.1001789)
Supplement: S1 Fig — (PDF) [file pmed.1001789.s002.pdf]

Figure S1 - Histopathology

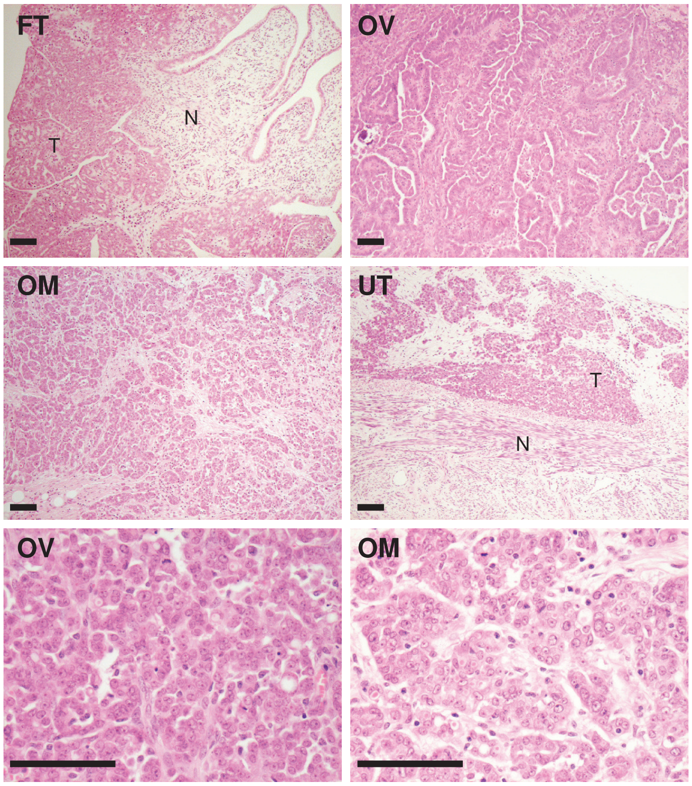

Figure 1: **Histopathology for case 8** showing uniform cellular architecture of high-grade serous ovarian carcinoma (HGSOC). H&E stained sections from interval debulking surgery specimens are shown, including right distal fallopian tube, right ovarian mass, omental metastasis and a serosal deposit on the uterus. The fallopian tube shows an invasive high-grade serous tubal carcinoma which is the presumed primary site for this case. A representative image of the omental metastases is shown as similar morphology and growth pattern was seen in the 3 blocks sampled. High-power fields are shown at bottom for ovarian and omental metastases showing conserved cellular architecture. FT, fallopian tube; OV, ovarian mass; OM, omental metastasis; UT, metastasis to uterine serosa; T, tumor; N, normal. Bars indicate 100μm.
