# Supplementary material for: Spatial and Temporal Heterogeneity in High-Grade Serous Ovarian Cancer: A Phylogenetic Analysis
Source: PLoS Med. 2015 Feb 24;12(2):e1001789. doi: 10.1371/journal.pmed.1001789 (PMC4339382; doi:10.1371/journal.pmed.1001789)
Supplement: S2 Fig — (PDF) [file pmed.1001789.s003.pdf]

Figure S2 - Cross-patient LOH frequencies

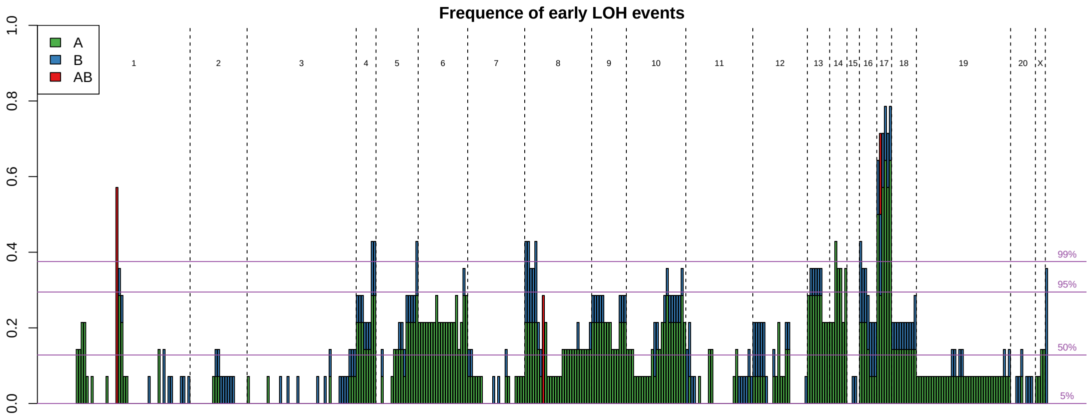

Figure 2: **Cross-patient loss-of-heterocygosity (LOH) events:** Frequency of LOH events across all patients at the time of the last common ancestor. We see enrichments above the simulated 99% (per-site) confidence bound on 4q, 5q and 8p and 17 (see main text).
