# Supplementary material for: Spatial and Temporal Heterogeneity in High-Grade Serous Ovarian Cancer: A Phylogenetic Analysis
Source: PLoS Med. 2015 Feb 24;12(2):e1001789. doi: 10.1371/journal.pmed.1001789 (PMC4339382; doi:10.1371/journal.pmed.1001789)
Supplement: S9 Fig — Caption as for S3 Fig. (PDF) [file pmed.1001789.s010.pdf]

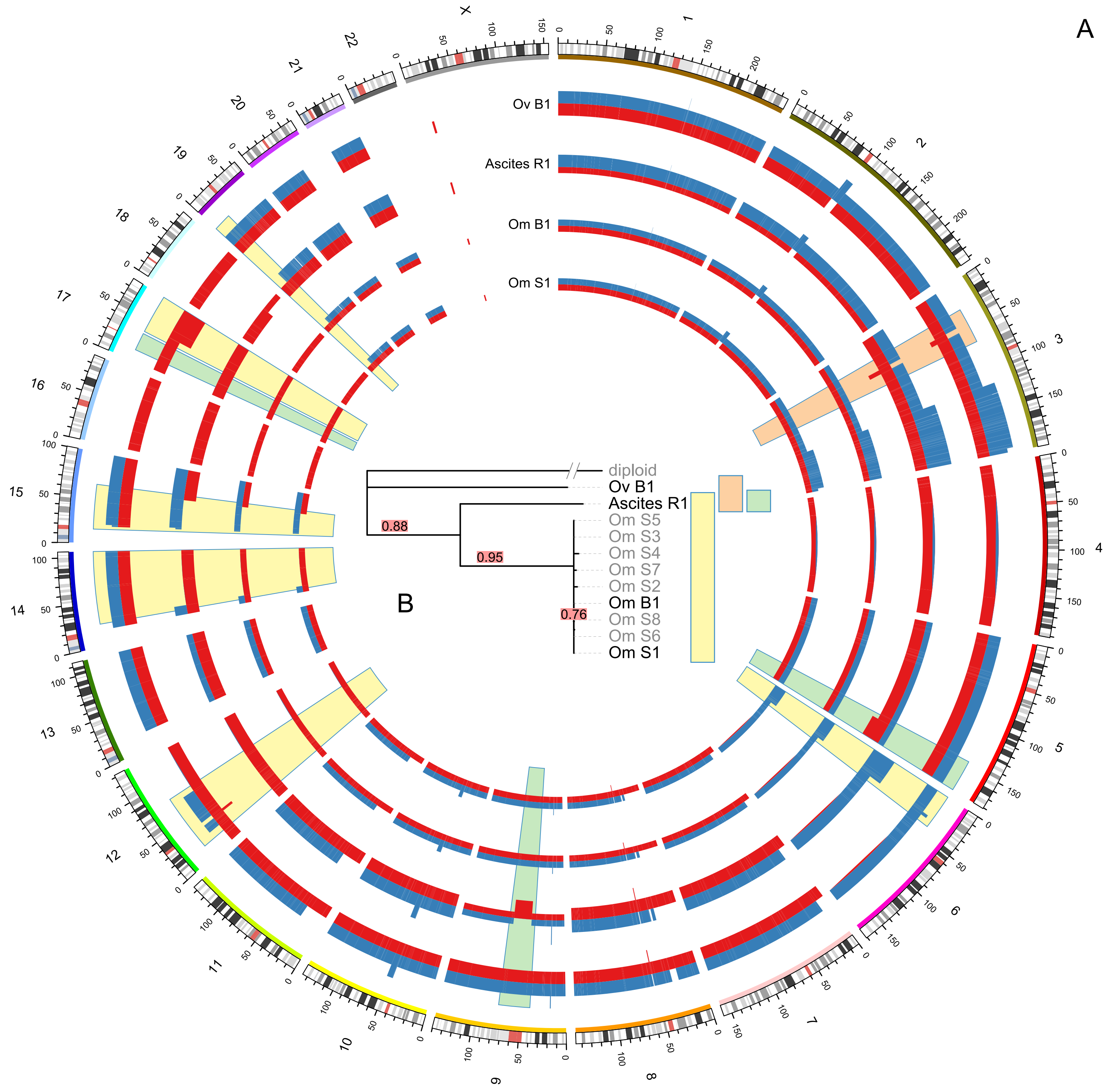

Figure 9: **Copy-number (CN) profiles (A) and evolutionary tree (B) for case 8.** Only selected CN profiles are shown and marked in bold in the evolutionary tree. Individual alleles are colored in red and blue. Confidence values for each split in the tree are given in red boxes. The color bars to the right of the tree and in the CN profiles indicate genomic events shared between *biopsy* and *relapse* samples (orange), *relapse* and *surgery* samples (yellow) and those that are unique to the *relapse* (green). Branch lengths are give in number of rearrangement events. Particularly long branches are marked in green.
