# Supplementary material for: Spatial and Temporal Heterogeneity in High-Grade Serous Ovarian Cancer: A Phylogenetic Analysis
Source: PLoS Med. 2015 Feb 24;12(2):e1001789. doi: 10.1371/journal.pmed.1001789 (PMC4339382; doi:10.1371/journal.pmed.1001789)
Supplement: S11 Fig — Caption as for S3 Fig. (PDF) [file pmed.1001789.s012.pdf]

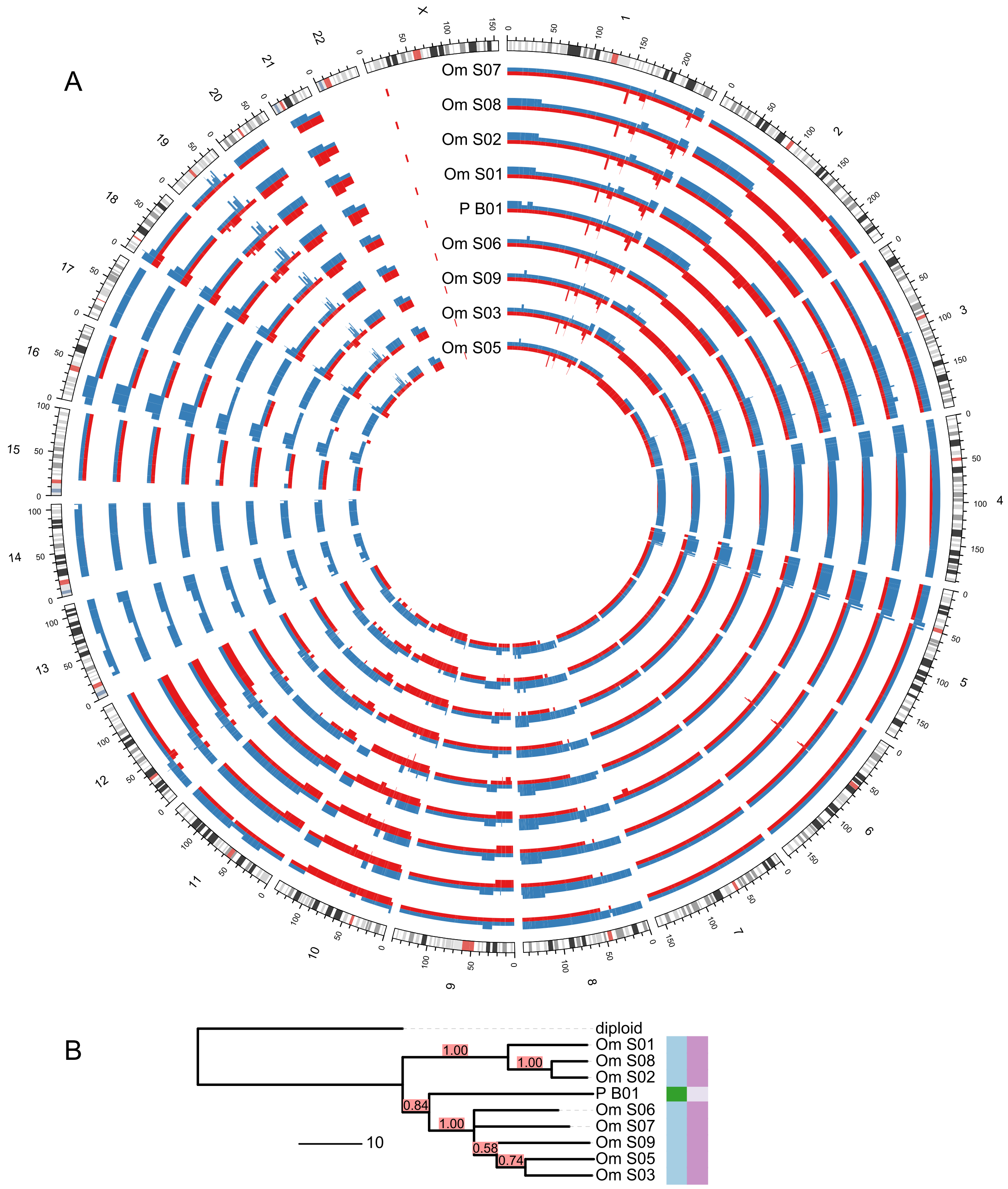

Figure 11: **Copy-number (CN) profiles (A) and evolutionary tree (B) for case 10.** Only selected CN profiles are shown and marked in bold in the evolutionary tree. Individual alleles are colored in red and blue. Confidence values for each split in the tree are given in red boxes. The color bars to the right of the tree indicate different sampling sites (left) and sampling times(right). Branch lengths are give in number of rearrangement events.
