# Supplementary material for: Spatial and Temporal Heterogeneity in High-Grade Serous Ovarian Cancer: A Phylogenetic Analysis
Source: PLoS Med. 2015 Feb 24;12(2):e1001789. doi: 10.1371/journal.pmed.1001789 (PMC4339382; doi:10.1371/journal.pmed.1001789)
Supplement: S17 Fig — (PDF) [file pmed.1001789.s018.pdf]

Figure S17 - Tree shapes and evolutionary patterns in resistant and sensitive cases

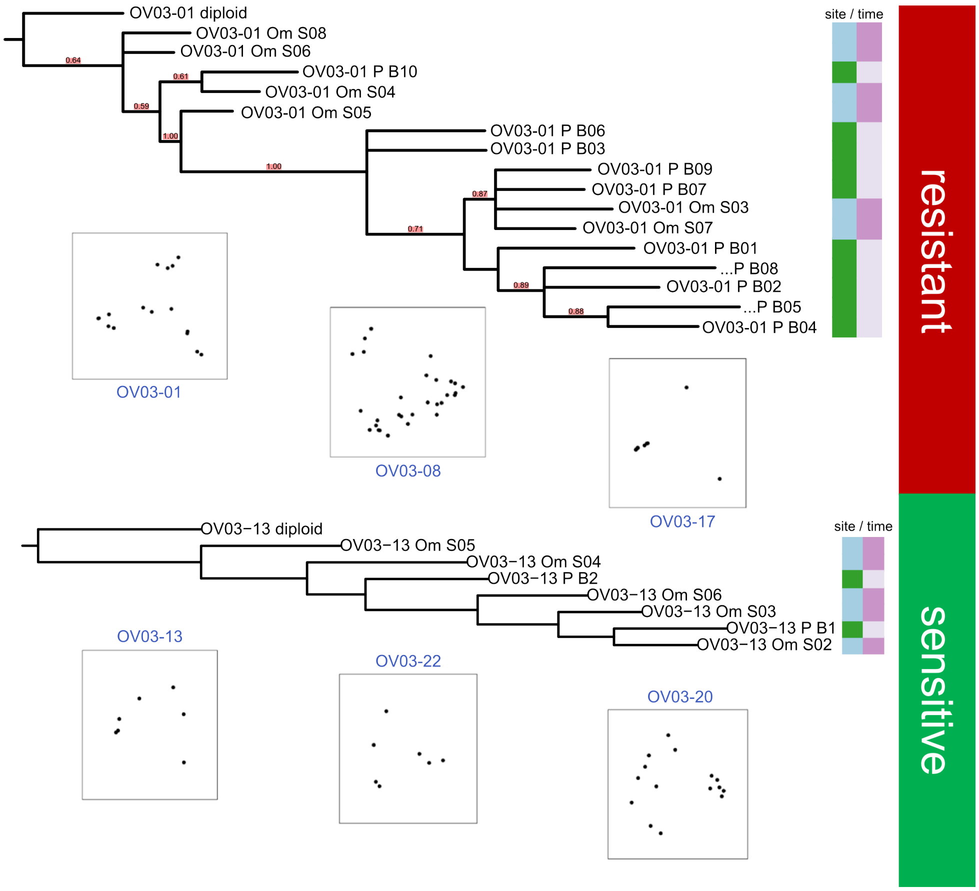

Figure 17: **Tree shapes and evolutionary patterns in resistant and sensitive cases.** Heavy clonal expansion, visible in clear clusters of samples in the scatterplots and high confidence support at multi-furcating internal branches (top), together with many changes in the course of chemotherapy (TD index, not shown) indicate poor outcome. In contrast, neutral-like evolutionary changes, indicated by constant branch lengths and few clonal expansions are indicative of better prognosis (bottom).
