# Supplementary material for: Spatial and Temporal Heterogeneity in High-Grade Serous Ovarian Cancer: A Phylogenetic Analysis
Source: PLoS Med. 2015 Feb 24;12(2):e1001789. doi: 10.1371/journal.pmed.1001789 (PMC4339382; doi:10.1371/journal.pmed.1001789)
Supplement: S18 Fig — (PDF) [file pmed.1001789.s019.pdf]

Figure S18 - Paired end sequencing of selected samples

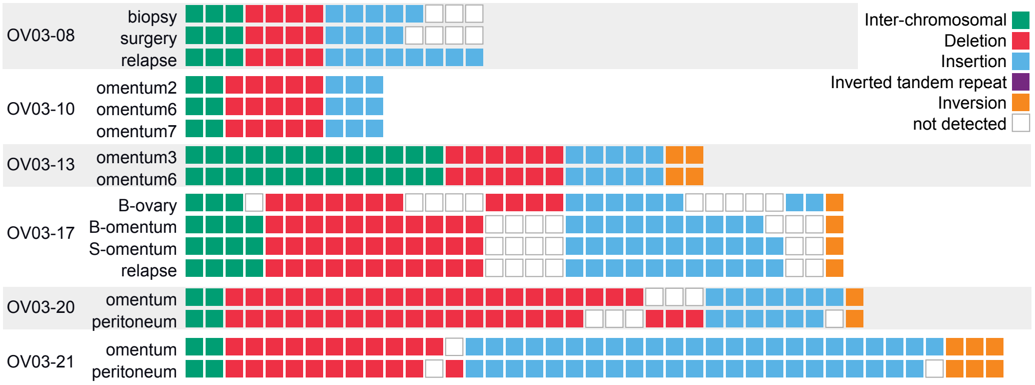

Figure 18: **Paired-end sequencing confirms ITH of selected samples.** We confirmed the experimental evidence for ITH from aCGH by paired-end sequencing. Within each patient, each column corresponds to a specific position on the genome. Filled and outlined rectangles indicate variable presence of specific breakpoints within samples from different cases. The breakpoints presented here are CN modifying breakpoints that were also found in the aCGH dataset, using a conservative validation scheme (see Methods).
