# Supplementary material for: Spatial and Temporal Heterogeneity in High-Grade Serous Ovarian Cancer: A Phylogenetic Analysis
Source: PLoS Med. 2015 Feb 24;12(2):e1001789. doi: 10.1371/journal.pmed.1001789 (PMC4339382; doi:10.1371/journal.pmed.1001789)
Supplement: S1 Table — (PDF) [file pmed.1001789.s021.pdf]

Table S1: Distribution of samples

	Arrays	Biopsy				Surgery				Relapse
		Ov/FT	Om	P	Others	Ov/FT	Om	P	Others	Ascites
1	16/20			10			6			
2	3/5				3					
3	18/20				4		14			
4	1/3			1						
5	29/29		2			1	20		5	1
6	8/8		1				7			
7	7/8			2		1	4			
8	11/11	1	1				8			1
9	15/16		3				5	1	6	
10	9/11			1			8			
11	7/17	1	4		1	1				
12	1/4		1							
13	3/3		1		2					
14	3/4				1	1	1			
15	3/5					3				
16	1/3		1							
17	3/4					1	2			
total# 17	138 / 171	2	14	14	11	8	75	1	11	2

Table 1: **Distribution of samples from the OV03/OV04 clinical studies across sites and time of sampling.** Table shows eligible patients recruited to the OV03/OV04 clinical studies and the number of samples available for genomic studies collected from various metastatic sites and at biopsy, interval debulking surgery and at relapse. Ov: Ovary; FT: Fallopian tube; Om: Omentum; P: Peritoneum.
