# Supplementary material for: Spatial and Temporal Heterogeneity in High-Grade Serous Ovarian Cancer: A Phylogenetic Analysis
Source: PLoS Med. 2015 Feb 24;12(2):e1001789. doi: 10.1371/journal.pmed.1001789 (PMC4339382; doi:10.1371/journal.pmed.1001789)
Supplement: S2 Table — (PDF) [file pmed.1001789.s022.pdf]

Table S2: Digital PCR results

Case	Mutant	Tissue	Type	Gene count	TP53 mutant	mutant gene/mutant TP53	Cellularity	WT TP53	mutant TP53 AF
8	NF1	Biopsy Site-B01	FFPE	0	131	0.00	NA	28	0.82
8	NF1	Omentum-S01	FFPE	86	87	0.99	60	114	0.43
8	NF1	Omentum-S02	FFPE	96	131	0.73	70	167	0.44
8	NF1	Omentum-S03	FFPE	260	260	1.00	70	327	0.44
8	NF1	Uterus-S01	FFPE	102	46	2.22	50	528	0.08
8	NF1	Left ovary-S01	FFPE	10	126	0.08	80	92	0.58
8	NF1	Uteru surface-S01	FFPE	81	97	0.84	80	411	0.19
8	NF1	Right ovary-S01	FFPE	21	178	0.12	80	927	0.16
8	NF1	Left fallopian tube-S01	FFPE	3	242	0.01	80	187	0.56
8	NF1	Left ovary-S01	FFPE	10	225	0.04	80	300	0.43
8	NF1	Ovary-B01	fresh	57	1120	0.05	80	273	0.80
8	NF1	Omentum-B01	fresh	218	825	0.26	70	1204	0.41
8	NF1	Omentum-S01	fresh	43	175	0.25	40	221	0.44
8	NF1	Omentum-S02	fresh	67	78	0.86	70	79	0.50
8	NF1	Omentum-S03	fresh	92	104	0.89	80	108	0.49
8	NF1	Omentum-S04	fresh	73	125	0.59	80	162	0.44
8	NF1	Omentum-S05	fresh	162	162	1.00	70	133	0.55
8	NF1	Omentum-S06	fresh	144	173	0.83	70	181	0.49
8	NF1	Omentum-S07	fresh	126	183	0.69	90	168	0.52
8	NF1	Omentum-S08	fresh	102	163	0.63	90	152	0.52
8	NF1	Ascites-R01	fresh	274	324	0.85	80	1	1.00
8	NF1	Buffy coat	fresh	0	0	0.00	NA	550	0.00
14	BRCA2	Omentum-S01	FFPE	39	67	0.58	50	260	0.20
14	BRCA2	Omentum-S02	FFPE	32	58	0.55	50	185	0.24
14	BRCA2	Omentum-S03	FFPE	51	57	0.89	60	119	0.32
14	BRCA2	Parametrium-S01	FFPE	26	42	0.62	50	371	0.10
14	BRCA2	Right ovary-S01	FFPE	31	56	0.55	70	358	0.14
14	BRCA2	Right ovary-S02	FFPE	35	54	0.65	70	203	0.21
14	BRCA2	Left ovary-S01	FFPE	0	15	0.00	50	1213	0.01
14	BRCA2	Left ovary-S02	FFPE	0	5	0.00	10	732	0.01
14	BRCA2	Left ovary-S03	FFPE	0	16	0.00	20	792	0.02
14	BRCA2	Unk-B01	fresh	79	85	0.929	NA	60	0.59
14	BRCA2	Left ovary-S01	fresh	35	42	0.83	NA	200	0.17
14	BRCA2	Omentum-S01	fresh	39	87	0.45	NA	354	0.20
14	BRCA2	Omentum-S02	fresh	30	104	0.29	10	837	0.11
14	BRCA2	Buffy coat	fresh	0	0	0.00	NA	203	0.00

Table 2: **Digital PCR results for *NF1* and *BRCA2* mutations** in HGSOC tissues from cases 8 and 14. AF, allele fraction; FFPE, formalin-fixed paraffin embedded; WT, wild-type
