# Supplementary material for: Spatial and Temporal Heterogeneity in High-Grade Serous Ovarian Cancer: A Phylogenetic Analysis
Source: PLoS Med. 2015 Feb 24;12(2):e1001789. doi: 10.1371/journal.pmed.1001789 (PMC4339382; doi:10.1371/journal.pmed.1001789)
Supplement: S3 Table — (PDF) [file pmed.1001789.s023.pdf]

Table S3: Primers for digital PCR

Primer name	Sequence
TP53_V216L_F	TGTGGAGTATTTGGATGACAGAAAC
TP53_V216L_R	ACCTCAGGCGGCTCATAGG
BRCA2_L2732X_F	GCCATTATTGAACTTACAGA
BRCA2_L2732X_R	CTGCCATTCTTTAAGACAG
BRCA2_L2732X_T_6FAM	[6FAM]AAGGCCCAGTAAGATCCTCCC[BHQ1]
BRCA2_L2732X_N_HEX	[HEX]AAGGCCCAGTTAGATCCTCCC[BHQ1]
TP53_V216L_T_VIC	[VIC]CTTTTCGACATAGTTTG
TP53_V216L_N_FAM	[FAM]CTTTTCGACATAGTGTGGT
NF1_deletion_F	TTTTGTTTACGAGCACAGATAACC
NF1_deletion_R	GAAACAGAAGATGACAGCAAAGAA

Table 3: **Primers used for Digital PCR experiments** with Fluidigm Biomark microfluidic system
