# Supplementary material for: Spatial and Temporal Heterogeneity in High-Grade Serous Ovarian Cancer: A Phylogenetic Analysis
Source: PLoS Med. 2015 Feb 24;12(2):e1001789. doi: 10.1371/journal.pmed.1001789 (PMC4339382; doi:10.1371/journal.pmed.1001789)
Supplement: S4 Table — (PDF) [file pmed.1001789.s024.pdf]

Table S4: CNA feature selection results

Region index	Chromosome	Start (bp)	End (bp)	Cytoband
316	chr15	58643988	100286564	q22.2, q22.31, q22.32, q22.33, q23, q24.1, q24.2, q24.3, q25.1, q25.2, q25.3, q26.1, q26.2, q26.3
222	chr9	127883987	140211216	q33.3, q34.11, q34.12, q34.13, q34.2, q34.3
221	chr9	112816993	127883306	q31.3, q32, q33.1, q33.2, q33.3

Table 4: **CNA feature selection results.** Genomic positions of the three most influential regions that separate resistant from sensitive patients as selected by the elastic net.
