# Supplementary material for: Spatial and Temporal Heterogeneity in High-Grade Serous Ovarian Cancer: A Phylogenetic Analysis
Source: PLoS Med. 2015 Feb 24;12(2):e1001789. doi: 10.1371/journal.pmed.1001789 (PMC4339382; doi:10.1371/journal.pmed.1001789)
Supplement: S1 Text — (PDF) [file pmed.1001789.s025.pdf]

Text S1: Case-by-case study of the OV03/04 cohort

Case 1

16 out of 20 samples remained after QC, 10 at initial biopsy and 6 at IDS. 6 Samples were from omentum and 10 from the peritoneum. Reconstruction of evolutionary history revealed a strong non-clock like pattern and a high degree of clonal expansion (CE index 1.26) confirmed by the high support values in the tree (Fig. S3B). Overall divergence was at 158 events from a hypothetical diploid (0.5σ above the cohort median), the fourth highest in the cohort and so was the TH index of 4.78.

The patient showed heterogeneity to an extent that clusters a subset of samples (S08, S06, B10, S04, S05) close to the root of the tree together with samples from a second patient in the supertree based on total CN (Fig. 1B in main text). This might suggest a rate-changing event on the long branch separating the subclades (marked in green, Fig. S3B) associated with an endoreduplication along this branch (compare top three tracks Fig. S3A with the rest). We further find large LOH events on chromosomes 4,6,8,9 and 17. The non-endoreduplicated subgroup also lost one allele on chr 15. We found a median of 120 events across all pairwise comparisons within that patients, the third highest in the patient cohort.

Case 2

3 out of 5 samples remained after QC, all of which were from biopsy and included one pelvis and two specified as “adnexa”. The tree was very clock-like and well supported with spatial heterogeneity indicated by the two adnexal sites clustering together (Fig. S4B). TH and CE were not measurable due to only three samples all of which were biopsy. The patient showed significant divergence from a hypothetical diploid of 146 events, but only a median of 45 events in all pairwise comparisons. The circos plot (Fig. S4A) confirms that within patient heterogeneity is relatively low. All samples had large LOH events on chromosomes 6, 8, 13, 14, 17 and 21.

Case 3

18 out of 20 samples remained after QC, out of which there were 14 omentum samples, 2 samples from the vaginal vault (VV) and two samples from bladder. The bladder and VV samples were taken at biopsy, all other were taken at IDS. This is another case of a non-clock like tree with a strongly supported branch (Fig. S5 green) that seems to have increased the mutation or proliferation rate. The tree shows a high degree of clonal expansion (CE index 1.24) and good support values for the omental and bladder/VV subclades. MEDICC scored counted 239 events to the hypothetical diploid and a median of 156 between all pairwise comparisons, both the respectively highest values in the study. The patient showed strong temporal heterogeneity (TH index 3.78).

When comparing genomic profiles between S01 and the other samples it seems to be again chromosomal amplifications that give rise to this split. For example, chromosomes 6,8,11 and 14 seem to have undergone whole chromosome duplications and there was a heterozygous loss of chromosome 15. We further detected large LOH events on chromosomes 4,5,9,10,13,14,16 and 17.

Case 4

Only 1 out of 3 samples remained after QC. Therefore case 4 was excluded from further analysis.

Case 5

All 29 samples remained after QC, out of which 22 were omentum samples. Out of these 2 were biopsy samples, all remaining samples were from IDS. Other sites sampled include right ovary, right paracolic gutter, small bowel mesentry and a relapse sample from ascites. The tree is clock-like with good support for the major splits but weak resolution in the major surgery clade. Clonal expansion is strong with good divergence between the main surgery clade and the biopsy/relapse group (CE index 1.47, Fig. S6B). Overall divergence was high with 195 events towards a hypothetical diploid and 133 events in median between all samples, the second highest in this study. Temporal heterogeneity was moderate (TH index 3.84).

As one of the two cases where relapse samples were available placement of the ascites clone was the primary focus. Similar to our observation in case 8 the relapse branches early after the first biopsy and shows clear signs of divergent evolution. This is evidenced by events such as deletion of remaining sections of allele A (blue) on 15q and 18q. The close relationships between the relapse and the biopsy can be seen on duplication events on 1p and 13q.

Case 6

All 8 acquired samples were of acceptable quality, all of which were from omentum. One sample was taken at biopsy the remaining 7 at IDS. The tree has a very clock like structure and showed signs of clonal expansion (CE index 0.74). Interestingly the biopsy sample is the outgroup for all other omental samples with a long branch indicating the strong temporal heterogeneity, i.e. strong genomic change in the course of therapy (TH index 6.66). Overall the patient showed long branches with 231 events in median from the hypothetical diploid, the second largest value in this study. The median of the pairwise distances is with 101 again suprisingly high given that all but one samples are taken at IDS from proximate sites.

All samples showed large LOH events on chromosomes 5, 9, 17 and 18. Comparison of genomic profiles pre- and post chemotherapy show that the major karyotype was already

established before therapy, with differences between the two groups being due to small segmental deletions, e.g. on 1p and 1q as well as 9q.

Case 7

7 out of 8 inital samples remained after QC, 2 of which were taken at biopsy, the others at IDS. All biopsy samples were from the peritoneum, all IDS samples from omentum. The tree fits a clock like model of evolution with only little clonal expansion (CE index 0.68, Fig. S8B). Overall this case showed little genomic change compared to other cases in the study with a median of only 60 events from a hypothetical diploid and a median of 65 events across all pairwise comparisons. In agreement with this observation, temporal heterogeneity was low (TH index 3.03) with the biopsy or surgery samples not forming distinctive subclades in the tree.

Large LOH events are seen on chromosomes 5,13,15,17 and 20. Interestingly, biopsy sample B02 still contained two copies of TP53 and BRCA1 which subsequently were lost. In general little genomic change can be seen when comparing profiles (Fig. S8A) at this resolution with visible events evenly spread between the biopsy and surgery samples, reiterating the low degree of temporal heterogeneity in this patient.

Case 8

All 11 samples remained after QC, with two biopsy samples from omentum and the right ovary, 8 omental samples taken at IDS and 1 relapse sample from ascites. The tree is very clock like with a remarkably homogenous clade of omentum samples that itself is divergent from both the ovary biopsy and the relapse (Fig. S9B). This impressive clonal expansion (CE index 2.22) comes with an overall small distance from the hypothetical diploid of only 25 events, the smallest value in the study. The patient also shows the smallest across sample divergence of only 3 events in the median. Because biopsy samples are present in the omentum clade as well as outside of it, temporal heterogeneity is low with a TH score of 3.46, the second smallest in the study.

In case 8 it was of special interest to verify the position of the relapse in the phylogenetic tree. We marked regions that indicate similarity to the omental subclade or the ovary outgroup in yellow and orange respectively (Fig. S9A). Apart from a LOH event on chr3 the major indicator for relatedness to the ovary biopsy is an apparent endoreduplication. Validation of the arrays by inspection of the raw intensity plots led us to believe that this is rather due to missegmentation then a real genomic event, which is in line with all other breakpoints (yellow) that support the phylogeny as given in Fig. S9B).

Case 9

15 out of 16 samples remained after QC. This included 8 omentum samples (two from biopsy), 5 SBM samples (all from IDS) as well as one peritoneum sample and one sample from the right paracolic gutter (both IDS). The tree is weakly non-clock like and shows

clonal expansions (CE index 0.65, Fig. S10B) tightly linked to the collection site: All SBM and Om samples form monophyletic groups with peritoneum and RPG as the respective outgroups. This significant spatial heterogeneity is on a background of 138 events from a hypothetical diploid which is average for this study. Biopsy samples cluster in the omental subclade, leading to a mediocre TH index of 4.54.

The circos plot (Fig. S10A) shows events that lead to the separation of groups, e.g segmental deletions on chromosomes 2 and 3 and amplifications on chromosomes 9 and 10. Again LOH events spanning the whole chromosomes across all samples are found on chromosomes 5, 10, 13 and 17.

Case 10

9 out of 11 samples remained after QC. Apart from one biopsy sample from peritoneum all remaining samples were omentum samples taken at IDS. The tree appeared clock-like with moderate clonal expansion (CE index 0.87, Fig. S11B). Overall divergence was average with a median of 128 events from a hypothetical diploid and 73 events in median between samples. Again there is clear evidence of spatial heterogeneity, i.e. clustering by site, in that the long peritoneum branch forms the outgroup of one of the two omentum subclades. This long branch also leads to a relatively high temporal heterogeneity score of 4.77 as peritoneum was the only biopsy sample.

Large LOH events affect chromosomes 4,10,13,14 and 17. Divergent evolution of the peritoneum sample can be seen especially on more focal events, such as a small homozygous deletion on 8p and a focal amplification on 10p (Fig. S11A).

Case 11

7 out of 17 samples remained after QC, 6 of which were taken at biospy. 4 were from omentum, one from the pelvis and two samples were from the left ovary, one at biopsy and the other at IDS. The tree showed clock-like structure with weak signs of clonal expansion (CE index 0.48, Fig. S12B), which might be suffering from the small number of remaining samples. Overall divergence was average with 137 events from a hypothetical diploid and a median of 95 events between samples. The surgery sample has a relatively short branch compared to the biopsy samples, leading to a high temporal heterogeneity index of 5.77.

This cases provided the unique chance to compare ovary samples pre and post chemotherapy. Interestingly we found a small CN neutral LOH event on 5q and a focal LOH deletion on 12q in the biopsy sample which could not have been regained in the surgery sample (Fig. S12A). This suggests that the surgery clone was a low prevalence subclone at biopsy. Comparison with the Cancer Gene Census showed that the 5q region contains the interleukin 6 signal transducer *IL6ST*. The surgery clone additionally acquired a small amplification on 10q and LOH on 14q. The biopsy clone additional amplified a 1.8mb region at the beginning of 15q.

Case 12

Out of 4 samples only 1 remained after QC. Therefore evolutionary analysis and assessment of heterogeneity was not possible and the patient was excluded from further analyses.

Case 13

All 3 samples remained after QC for this case, all of which were taken at biopsy, one from the diaphragm, pelvis and omentum each. The clock-like tree shows a clear separation of the omentum sample from the two others (Fig. S13B). Overall divergence was low with a median of 64 events towards a hypothetical diploid and a median of 34 events between samples. Temporal heterogeneity could not be measured as all samples were taken at biopsy. Clonal expansion is detectable (CE 0.62) but needs to be evalued carefully given the small sample size.

All samples had undergone endoreduplication and large scale LOH on chromosomes 4,5,6,16,17,18 and 19. The omentum sample differes from the pelvis and diaphragm by retaining segments of the second allele (red) of chromosome 4p, 6p and 16q (Fig. S13A).

Case 14

3 out of 4 samples remained after QC with two IDS samples taken from omentum and the left ovary and one biopsy sample of unknown origin. The tree is highly non-clock like with one long branch separating the biopsy sample from the others (Fig. S14B). Despite this strong signal relationship of the biopsy sample to the others could not be convincingly resolved. This is mainly due to the low overall divergence with only 24 events to a hypothetical diploid, the lowest score in the study. The median pairwise distance is equally low with 31 events, mainly due to the divergent evolution along the biopsy branch. This long branch gives rise to an elevated temporal heterogeneity score of 4.67, whereas clonal expansion is hardly detectable (CE 0.61).

Compared to the omentum and ovary sample which looked very diploid-like, the biopsy sample shows a highly rugged CN profile with additional LOH events on chromosomes 6,7,13,14,15,16 and 17 (Fig. S14). Notably this is one of the few cases where major parts of chr 17 are intact for the majority of samples, including segments harboring *TP53* and *BRCA1*.

Case 15

3 out of 5 samples remained after QC, all of which were from IDS. Two samples were from the right ovary and one sample from the left ovary. The tree is clock-like with a weakly supported split that groups one of the right ovary samples with the left ovary, leaving the second right ovary sample as the outgroup (Fig. S15B). Overall divergence was low with a median of 66 events from a hypothetical diploid and a median across sample distance of

10 events, the second smallest in the study. No temporal heterogeneity could be measured as all samples were from IDS. Clonal expansion was detectable (CE index 0.74).

Large LOH events were found across all samples on chromosomes 4,5,6,16 and 17 (Fig. S15A). Small events favor a closer relationship between the two right ovary samples (e.g. deletion on the remaining allele of 17q).

Case 16

Only 1 sample remained of originally 3 after QC. Evolutionary analysis and assessment of heterogeneity was therefore not possible and the patient was excluded from further analyses.

Case 17

3 out of 4 samples remained after QC, all of which were from IDS. The set contained two omentum samples and one sample from the left ovary. The tree is clock like with reasonable support for the surprising split grouping the ovary with the second omentum sample (Fig. S16B). Large events such as LOH on chromosomes 9,15,16,17 and 18 support this hypothesis. Smaller events such as amplification on chromosome 8p contradict that. The relatively small overall divergence (94 events from a hypothetical diploid and a median of 81 events between samples) with the small sample size makes this question difficult to answer. Different allelic frequencies of different breakpoints might play an additional role. Temporal heterogeneity could not be determined as all samples were from IDS.
